# Co-Orientation: Quantifying Simultaneous Co-Localization and Orientational Alignment of Filaments in Light Microscopy

**DOI:** 10.1371/journal.pone.0131756

**Published:** 2015-07-10

**Authors:** Robert P. J. Nieuwenhuizen, Leila Nahidiazar, Erik M. M. Manders, Kees Jalink, Sjoerd Stallinga, Bernd Rieger

**Affiliations:** 1 Quantitative Imaging group, Department of Imaging Physics, Delft University of Technology, Delft, The Netherlands; 2 Cell Biophysics group, Department of Cell biology, The Netherlands Cancer Institute, Amsterdam, The Netherlands; 3 Van Leeuwenhoek Centre for Advanced Microscopy, Molecular Cytology, Swammerdam Institute for Life Sciences, University of Amsterdam, Amsterdam, The Netherlands; University of New South Wales, AUSTRALIA

## Abstract

Co-localization analysis is a widely used tool to seek evidence for functional interactions between molecules in different color channels in microscopic images. Here we extend the basic co-localization analysis by including the orientations of the structures on which the molecules reside. We refer to the combination of co-localization of molecules and orientational alignment of the structures on which they reside as co-orientation. Because the orientation varies with the length scale at which it is evaluated, we consider this scale as a separate informative dimension in the analysis. Additionally we introduce a data driven method for testing the statistical significance of the co-orientation and provide a method for visualizing the local co-orientation strength in images. We demonstrate our methods on simulated localization microscopy data of filamentous structures, as well as experimental images of similar structures acquired with localization microscopy in different color channels. We also show that in cultured primary HUVEC endothelial cells, filaments of the intermediate filament vimentin run close to and parallel with microtubuli. In contrast, no co-orientation was found between keratin and actin filaments. Co-orientation between vimentin and tubulin was also observed in an endothelial cell line, albeit to a lesser extent, but not in 3T3 fibroblasts. These data therefore suggest that microtubuli functionally interact with the vimentin network in a cell-type specific manner.

## Introduction

Cytoskeletal protein networks serve a number of crucial roles in living cells. Traditionally, three types of cytoskeletal networks are discriminated [1]. First, thin filaments with a diameter of about 10 nm, which consist of actin polymers with associated cross-linking proteins and “muscle-like” myosins give stiffness to cells and play important roles in the generation of motile forces. Second, microtubules, which consist of hollow tubules of the protein tubulin with an outer diameter of approximately 23 nm. Microtubules run throughout the cell and play a dominant role as cellular highways for the transport of cargo, which can be moved either outwards from or inwards to the center of the cell by specific, ATP-consuming motor proteins. The third type of cytoskeleton are termed intermediate filaments due to their intermediate unit-filament diameter. Over 60 different proteins such as keratins, vimentin and lamins have been identified, most of which have a strict cell type-specific distribution. Whereas each of these filament systems, their subunits and methods of polymerization have been the subject of many thousands of studies, remarkably little is known on how the three principal filament systems may interact and collaborate to keep the cell alive and functioning. This is due in part because imaging with confocal fluorescence microscopy provides insufficient resolution to reliably discriminate individual filaments in most cases, whereas electron microscopy does provide ample resolution but is much less suited to routinely identify and track the different filaments. The recent advances in optical super-resolution microscopy, including localization microscopy [2–6] and STED microscopy [7] do provide sufficient resolution to distinguish individual fluorescently labeled filaments within the cell, and they can be routinely applied in a convenient manner.

The availability of superresolved multicolor images of filaments introduces the need for new quantitative tools to interrogate the organization of and mutual interrelations between the different cytoskeletal elements. Tools developed for diffraction limited fluorescence microscopy focused on the problem of co-localization analysis. This analysis asks whether images show evidence for possible interactions between the molecules imaged in both color channels. Typically the answer to this question is expressed in terms of: 1) the Pearson correlation coefficient between the intensities [8]; 2) the Manders coefficients, which are defined as the fraction of the total intensity per channel that occurs in co-localizing pixels [9], i.e. pixels whose values in both channels exceed certain thresholds; or 3) the overlap fractions of segmented objects in both color channels [10].

The different measures of co-localization cannot simply be applied to localization microscopy techniques; these techniques produce datasets consisting of coordinates of localized molecules instead of intensity values in pixels. This suggests that coordinate based analyses of distances between molecules should be used instead. Proposed measures include: the pair-correlation function between coordinates in two color channels [11]; a hypothetical potential energy function that is estimated from the distances from each localization to the nearest neighbor in the other color channel [12]; and the rank correlation between the distances from a localization to its neighbors in the same color channel on the one hand and distances to its neighbors in the other channel on the other hand [13]. However, all these analyses only consider the spatial proximity of molecules in different color channels. They do not take into account that the molecules reside on extensive structures such as filaments that have additional geometric features such as size, orientation or curvature.

Here we report a rigorous quantitative framework for analyzing the simultaneous co-localization and similarity in orientation of structures in multicolor images. We will refer to the combination of co-localization and orientational alignment as co-orientation. We focus here on the orientation as a geometric feature as it presents a particularly salient property of cytoskeletal filament networks. Because the orientation varies with the length scale at which it is evaluated, we include this scale as a separate informative dimension for the analysis. We demonstrate our methods on simulated localization microscopy data of filament structures, as well as experimental images of filamentous structures acquired with localization microscopy in different color channels. Software for our co-orientation analysis is freely available in the form of Matlab code at http://www.diplib.org/add-ons/.

## Materials and Methods

### Orientation measurement

The co-orientation analysis starts with the determination of the orientation in each color channel. The two images of two different molecular species imaged in color channels *l* = 1, 2 will be denoted with $I_{l}(\vec{x})$. For now we will assume these to be two-dimensional and we will discuss the generalization to three-dimensional images below. In this work we will only apply our methods to localization microscopy data. The estimated fluorophore coordinates are converted into images by binning them into two-dimensional histogram with bin sizes of 10 nm. It should be noted here that although all subsequent operations are carried out on pixelated images, this is not problematic when the pixel size is smaller than 1.5 times the localization precision [14] because the information lost at small length scale is limited. For smaller pixel sizes we do not expect that the choice of pixel size affects any outcomes. Note also that in principle rendering localizations as Gaussian blobs the size of the localization error distribution provides a better data representation than the histogram binning applied here [15]. However, in practice this rendering is too slow due for the large number of required renderings for the significance tests that are discussed below.

The orientations of the filaments in the images are analyzed by considering orientation space representations $I_{l}(\vec{x},\phi )$ [16], which quantify for each position $\vec{x}$ how much evidence there is for the presence of structures with an orientation *ϕ*. By considering multiple orientations, it is possible to determine the orientations of several crossing filaments at the same location.

To compute $I_{1}(\vec{x},\phi )$ and $I_{2}(\vec{x},\phi )$, the images $I_{1}(\vec{x})$ and $I_{2}(\vec{x})$ are first filtered with a set of orientation selective filters $\Phi \left(\vec{x};\phi \right)$, which have an orientation *ϕ* between −*π*/2 and *π*/2 with respect to the x-axis. Applying these filters gives the orientation space representation:

$$I_{l}(\vec{x},\phi )=I_{l}(\vec{x})*\Phi (\vec{x};\phi )\;,$$ (1)

where * denotes the convolution operation, and the filters $\Phi \left(\vec{x};\phi \right)$ are defined by their Fourier transforms:

$$\hat{\Phi }(\vec{q};\phi )=\int_{-\infty }^{\infty }\Phi (\vec{x};\phi )\;\exp\;\left(-i2\pi \vec{q}\cdot \vec{x}\right)d^{2}r\;,$$ (2)

as [17]:

$$\hat{\Phi }\left(\vec{q};\phi \right)\equiv 2\;\text{exp}\;(-\frac{{\left(\phi_{q}-\phi \right)}^{2}}{2w_{\phi }^{2}}){\left(qs_{o}\right)}^{w_{q}^{2}s_{o}^{2}}\text{exp}\left(-\frac{q^{2}s_{o}^{2}-1}{2w_{q}^{2}s_{o}^{2}}\right).$$ (3)

Here *ϕ*_*q*_ is the angle of $\vec{q}$ with respect to the x-axis, *w*_*ϕ*_ is the angular bandwidth of the filter, *s*_*o*_ is the length scale for which the orientation is evaluated and *w*_*q*_ is the bandwidth of the filter with respect to the spatial frequency magnitude $q=\left|\vec{q}\right|$. For this work we chose *w*_*q*_ = 0.8/*s*_*o*_ and the orientation scale *s*_*o*_ was determined by selecting the smallest value that still had a good orientation selectivity upon visual inspection of the orientation space representation. Generally, the scale should be set such that the features of interest have a high contrast with respect to the local background and a high contrast with respect to the responses at the same location to filters with different orientations. However, it does not make sense to choose a scale smaller than the resolution of the images [14]. The width *w*_*ϕ*_ is derived from the number of independent orientations *n*_*o*_ that are analyzed via *w*_*ϕ*_ = *π*/*n*_*o*_. Here we used *n*_*o*_ = 41 for simulated datasets and for experimental datasets, which gives an angular resolution of about 77 mrad. This is on the same order as the angular extent of linelike structures with a width *w* at a scale *s*_*o*_ which is *w*/*s*_*o*_ ∼ 0.05 (for *w* ∼ 10 nm and *s*_*o*_ = 200 nm). Note that by definition $I_{l}\left(\vec{x},\phi +\pi \right)=I_{l}\left(\vec{x},\phi \right)$.

Next, we take the absolute value of the orientation space representation and subtract the minimum value per location $\vec{x}$. Subsequently we normalize the outcome such that the sum over *ϕ* in each location equals the number of localizations by computing:

$${\tilde{I}}_{l}(\vec{x},\phi )=(\frac{\left|I_{l}(\vec{x},\phi )|-{\text{min}}_{\phi }(|I_{l}(\vec{x},\phi )|\right)}{\int_{-\pi /2}^{\pi /2}|I_{l}(\vec{x},\phi^{\prime })|d\phi^{\prime }-\pi \;{\text{min}}_{\phi }(|I_{l}(\vec{x},\phi )|)})I_{l}(\vec{x})\;.$$ (4)

${\tilde{I}}_{l}\left(\vec{x},\phi \right)$ can be interpreted as the expected density of localizations in channel *l* at position $\vec{x}$ belonging to molecules in filaments with local orientation *ϕ*. The subtraction of the minimum corrects for the non-zero response given by the filters $\Phi \left(\vec{x};\phi \right)$ for orientations that do not correspond to the orientations of the filaments at $\vec{x}$.

For three-dimensional images, the three-dimensional orientation can be analyzed in a similar manner, see e.g. [18]. The generalization of the normalization in Eq 4 for three-dimensional orientation space representation involves normalization over solid angles. However, the orientation difference can always be expressed as a single angle.

### Co-orientation analysis

**Fig 1:**
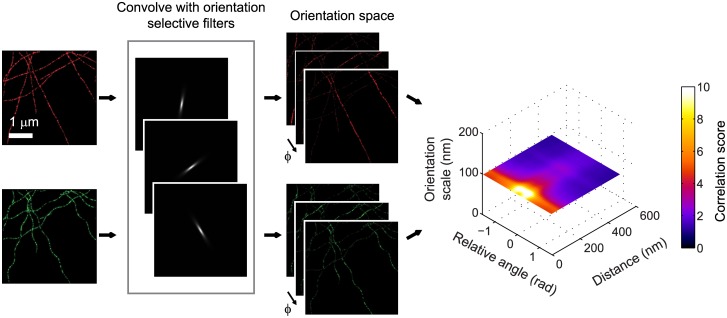
Steps for obtaining the co-orientation plot. To compute the co-orientation plot, the images in both color channels are first processed by a filter bank of orientation selective filters (shown here for an orientation scale of 100 nm). This provides orientation space representations of both channels with the evidence per orientation in each pixel. The cross-correlation between these representations then leads to the co-orientation plot showing the correlation *c* as a function of the distance between localizations and angle between the filaments they belong to.

The next step in the analysis is to define a measure that quantifies both the co-localization and orientational alignment of structures in the two color channels. For this purpose we extend the concept of the cross-correlation function used in localization microscopy [11] to the generalized cross-correlation function:

$$c\left(\Delta \vec{x},\Delta \phi \right)=\pi \frac{\langle {\tilde{I}}_{1}\left(\vec{x},\phi \right){\tilde{I}}_{2}\left(\vec{x}+\Delta \vec{x},\phi +\Delta \phi \right)\rangle }{\langle I_{1}\rangle \langle I_{2}\rangle },$$ (5)

where ⟨.⟩ denotes the averaging operation over both $\vec{x}$ and *ϕ*. The averaging over the spatial coordinate $\vec{x}$ is restricted to the selected region of interest, which typically excludes regions outside cells. The multiplication with *π* gives $c\left(\Delta \vec{x},\Delta \phi \right)=1$ for statistically independent images. Often it will be convenient to compute the average of $c\left(\Delta \vec{x},\Delta \phi \right)$ over circles of constant distance $\left|\Delta \vec{x}\right|=r$, which we will denote with *c*(*r*, Δ*ϕ*). An illustration of the steps needed to compute $c\left(\Delta \vec{x},\Delta \phi \right)$ from the superresolution images is shown in Fig 1.

The cross-correlation in $c\left(\Delta \vec{x},\Delta \phi \right)$ is efficiently computed using three-dimensional (*x*, *y*, *ϕ*) Fourier transformations:

$$c=\pi \frac{{\text{FT}}^{-1}\left(\text{FT}\;\left({\tilde{I}}_{1}\right)\;\text{FT}\;{\left({\tilde{I}}_{2}\right)}^{*}\right)}{\langle I_{1}\rangle \langle I_{2}\rangle {\text{FT}}^{-1}\;(|\text{FT}\left(W\right){|}^{2}\text{)}},$$ (6)

where *W* is a two-dimensional binary mask image that has a value of 1 inside the selected region of interest and 0 outside.

The interpretation of *c*(*r*, Δ*ϕ*) is as follows: for a typical point on a filament in one channel, it is the density of filaments in the other channel at a distance *r* with a relative orientation (i.e. angle with the first filament) of *ϕ* which is normalized by the density that would have been obtained if the filaments were statistically independent. Alternatively, it could also be interpreted as a normalized probability density for two randomly chosen points on two filaments in different color channels to have a separation *r* and an orientation difference *ϕ* between the filaments they belong to. Several examples to illustrate the interpretation of the co-orientation plot are shown in S1 Fig, S2 Fig and S3 Fig.

### Testing for statistical significance

A measure for the strength of the co-orientation in an image is given by the normalized anisotropic Ripley’s K statistic *K*_∥_(*R*), which is computed as:

$$K_{∥}\left(R\right)=\frac{1}{\pi R^{2}}\frac{2}{\pi }\int_{A}\int_{-\pi /2}^{\pi /2}d^{2}\;\Delta x\;d\Delta \phi \;c(\Delta \vec{x},\Delta \phi )\cos(2\Delta \phi )\;,$$ (7)

where *A* denotes a circular domain with radius *R*. The rationale for choosing a cos (2Δ*ϕ*) weight is the following: assuming that $c\left(\Delta \vec{x},\phi \right)$ is symmetric with respect to Δ*ϕ*, this weight returns the strength of the second nonzero term of a Fourier series expansion of $c\left(\Delta \vec{x},\phi \right)$. Therefore it expresses to first order the tendency of $c\left(\Delta \vec{x},\phi \right)$ to assume higher values for smaller angles Δ*ϕ*. Filaments with relative smaller angles contribute positively to *K*_∥_(*R*) whereas perpendicularly crossing filaments have a negative contribution. The first term in the same Fourier series expansion of $c\left(\Delta \vec{x},\phi \right)$ has a constant weight with respect to Δ*ϕ* and thus gives a result that is proportional to Ripley’s K statistic and expresses co-localization rather than co-orientation. The higher order terms in the Fourier series expansion could be used to describe more complicated relationships between the co-localization and orientations of filaments.

The anisotropic Ripley’s K statistic *K*_∥_(*R*) was used to test the statistical significance of the co-orientation of individual images. The radius *R* is chosen beforehand by the experimenter and expresses the range of the co-orientation effect. In theory, all possible radii *R* could be relevant and could all be tested, while keeping in mind that tests at different radii are not statistically independent. However, in practice this is unnecessarily complicated and a single radius *R* can be set such that the main peak in the co-orientation plot at small distances *r* is captured in the significance test. Alternatively, prior expectations about the range of physically meaningful effects can also be used to determine a single value of *R* for testing.

The null hypothesis for the significance test is that the filaments in both color channels do not interact and are thus statistically independent, which implies that the expected value of *K*_∥_(*R*) is 0. The expected deviations from 0 under the null hypothesis are very difficult to treat analytically due to the statistical dependencies between the localizations in each color channel [19]. These dependencies arise firstly because the localized molecules are constrained in their positions because they reside in filaments and secondly because each molecule is localized multiple times. Therefore we assume as a working assumption that under the null hypothesis, *K*_∥_(*R*) is normally distributed with a mean value of 0 and variance $\sigma_{K}^{2}$, which was estimated as follows. Firstly, a circular region of interest is selected in the images. Next, the image of the second color channel is rotated with respect to the image of the first color channel over equally spaced angles *θ* between 0 and 2*π*. Note that the ROI was chosen to be circular in order to ensure that the sum of pixel values in each channel does not change with the rotation. For each rotation we recomputed *K*_∥_(*R*), giving the co-orientation strength per rotation *K*_∥_(*R*; *θ*). The variance $\sigma_{K}^{2}$ was then computed as:

$$\sigma_{K}^{2}={\left(\frac{1}{n_{\theta }}\underset{\theta }{\sum }K_{∥}\left(R;\theta \right)\right)}^{2}+\frac{1}{2n_{\theta }}\underset{\theta }{\sum }{\left(K_{∥}\left(R;\theta \right)-K_{∥}\left(R;-\theta \right)\right)}^{2},$$ (8)

where *n*_*θ*_ is the number of angles *θ* (see S1 Text for a derivation). Given $\sigma_{K}^{2}$, the probability of having a value *K*_∥_(*R*) at *θ* = 0 under the null hypothesis is given by

$$P=\frac{1}{2}(1+\text{erf}(\frac{K_{∥}\left(R\right)}{\sigma_{K}\sqrt{2}}))\;,$$ (9)

where erf(.) denotes the error function.

Note that our method resembles the approach of Van Steensel et al. [20] for qualitatively determining if the co-localization in diffraction limited fluorescence imaging may be significant. In this approach the image in one color channel is shifted instead of rotated. Furthermore, it is important to note that $\sigma_{K}^{2}$ does not accurately predict the uncertainty in *K*_∥_(*R*) if the null hypothesis does not hold. Therefore it cannot be used to test differences in co-orientation strength between images. Instead, sets of values for *K*_∥_(*R*) obtained from several datasets representing one biological condition can be compared with another set of values representing another condition using standard statistical tests such as the Mann-Whitney U test [21].

### Local co-orientation

In order to detect which parts of a region of interest exhibit the strongest co-orientation, we developed a scheme for visualizing the local co-orientation strength. In this scheme we determine *K*_∥_(*R*) in square subregions of the image with a size of 3*R* which were displaced by multiples of *R* horizontally or vertically with respect to each other, i.e. two-thirds of the pixels in each region overlapped with two-thirds of the pixels in each adjacent region. For each subregion, we took the previously determined orientation space representations ${\tilde{I}}_{l}\left(\vec{x},\phi \right)$ and used it to compute $c\left(\Delta \vec{x},\Delta \phi \right)$, where the average densities ⟨*I*_*l*_⟩ across the field of view were used in the denominator rather than the averages per subregion. *K*_∥_(*R*) then follows from $c\left(\Delta \vec{x},\Delta \phi \right)$ as before.

To ensure a smooth visualization, the values of *K*_∥_(*R*) were assigned to the center point of each subregion and linearly interpolated in between these points. A visualization of the local co-orientation was then obtained by applying a blue overlay to the image of the filaments, where the negative pixel values were set to 0, the brightest 3% of the pixels were clipped and the remaining pixels were linearly scaled between 0 and 255. See S6 Fig for an example of how the percentage of clipped pixels affects the appearance of the overlay.

Note that in this visualization scheme, crossings of filaments lead to a low score for the local co-orientation strength which may be unintuitive in some cases. Instead, it is also possible to replace the cos(2*ϕ*) weight in the computation of *K*_∥_(*R*) in Eq 7 by a cos^2^(*ϕ*) weight. However, unlike with the cos(2*ϕ*) weighting, the cos^2^(*ϕ*) weighting also makes the score sensitive to mere co-localization without orientational alignment. Therefore it is generally best to compare images with both kinds of weighting for identifying areas with strong co-orientation.

A somewhat computationally faster method to approximate the local co-orientation strength can be implemented using convolution operations. Specifically, the orientation space representation ${\tilde{I}}_{1}$ has to be convolved with a kernel $g\left(\vec{x},\phi \right)=\text{cos}\left(2\phi \right)O\;\left(\vec{x}/R\right)$, subsequently multiplied by ${\tilde{I}}_{2}$ and summed over *ϕ*, followed by a smoothing with a kernel $O\;\left(\vec{x}/3R\right)$ and finally a multiplication by a normalization constant. Here the circular kernel $O\;\left(\vec{x}\right)=1$ if $\left|\vec{x}\right|<1$ and 0 otherwise.

### Simulations of test data

Simulated localization microscopy images in two color channels were obtained in two steps. Firstly, two-dimensional images of filaments were generated for both color channels. Secondly, positions of fluorescent molecules are generated and several localizations of each of these fluorophores were simulated.

The filaments in one color channel were generated according to the two-dimensional wormlike chain model of Kratky and Porod [22]: All filaments consisted of 10^4^ connected segments of 1 nm. The position of the central segment was randomly positioned within a circular region with a radius of $FOV\sqrt{2}+L/2$, where *FOV* = 4 *μ*m is the size of the field of view for the final image and *L* is the length of the filament. This circular region was deliberately chosen to be large enough to ensure a homogeneous and anisotropic distribution of filaments within the field of view. The orientation of the central segments was chosen randomly between −*π* and *π*. Angles between subsequent segments of the filament were taken from a normal distribution with standard deviation 1 nm/*ξ*, where *ξ* is the persistence length of the filament. The filaments in the second color channel were obtained in various manners: firstly by displacing each filament in the first channel over a fixed distance perpendicular to its orientation; secondly by independently simulating them in the same way as the filaments in the first channel but with a different persistence length; thirdly by displacing each segment perpendicular to their orientation with a sinusoidally modulated magnitude of the displacement such that the filaments in the second channel appeared to be twisted around those in the first channel. Finally, image representations of the filaments were made by counting the number of connecting points between segments in pixel bins of 5 nm in size, and convolving the resulting images with a Gaussian kernel with a full width at half maximum *FWHM* = 5 nm to account for the finite width of the filaments.

Subsequently, localization datasets were simulated from the images of the filaments. A Poisson distributed number of *N* fluorophores was obtained with a relative density proportional to the pixel values in the filament images. The positions of these fluorophores were then displaced with a Gaussian probability density with *FWHM* = 5 nm to account for the size of the antibodies linked to the fluorophores. Each fluorophore was then assigned a random number of localizations *M* defined as the minimum of two quantities: *M*_*poisson*_ and *M*_*geo*_ drawn from a Poisson distribution with an expected value of 25 and a geometric distribution with an expected value of 11 respectively. Localizations were then finally displaced with a Gaussian probability density with standard deviation *σ*, where a different value of *σ* was randomly generated for each localization based on the expression in Eq 4 in Ref. [23, 24] and using the following values: the number of signal photons per localization *n*_*ph*_ (drawn from a geometric distribution with an expected value of 2000), background photons *b* (average of 9 × 9 Poisson distributed values with expected value of 1), and the PSF width *σ*_*a*_ (Gaussian distributed with mean 0.3 × *λ*/*NA* = 0.3 × 670/1.45 ≈ 1.38 and standard deviaiton of 2% of the mean; this is roughly the distribution we obtain when fitting the PSF of Alexa Fluor 647 fluorophores and is in agreement with the range of previously suggested values [25]). All images in which the simulated datasets are visualized were obtained by rendering visualizations as Gaussian blobs with a kernel size equal to *σ*.

### Acquisition and processing of experimental data

#### Sample preparation

Primary human umbilical vein endothelial cells (HUVECs) were purchased from Lonza and cultured on fibronectin (Sanquin)-coated dishes in EGM-2 medium, supplemented with SingleQuots (Lonza) at 37°C and under 5% CO_2_ until passage 8. To stain vimentin and tubulin, HUVEC cells were grown for 24 hours on cleaned #1.5 coverslips in Medium 200 (Life technologies) with the addition of Low Serum Growth Supplement (LSGS) (Life technologies) at 5%.

Immortalized Human Vascular Endothelial Cells (EC-RF24) [26] were grown in a mixture of HUVEC cell medium, 25% DMEM and 25% RPMI. NIH-3T3 mouse fibroblasts were maintained in DMEM supplemented with 10% fetal calf serum (FCS) as previously described [27]. The cells then were fixed with 10% MeS buffer (100 mM MeS, pH 6.9, 1mM EGTA and 1mM MgCl_2_) and 90% methanol for 5 minutes on ice. After blocking with 5% Bovine Serum Albumin (BSA) for 1 hour, HUVEC and EC-RF24 cells were incubated with rabbit anti-tubulin polyclonal antibodies (Abcam) and mouse anti-vimentin monoclonal antibodies (Clone V9-Dako) for 1 hour. NIH-3T3 mouse fibroblasts were stained with anti-tubulin antibody raised in mouse (Sigma-Aldrich) and rabbit monoclonal antibody against vimentin (GeneTex). Subsequently all the cells were incubated with goat anti-rabbit and goat anti-mouse antibodies (Alexa 488, Alexa 647, Invitrogen) for 30 minutes. All the fixation and staining steps were done at room temperature. Control experiments were also performed where the fluorophore types labeling the secondary antibodies were swapped to rule out color-related artefacts.

In the case of actin and keratin, primary keratinocytes isolated from newborn (1–3 day old) plectin deficient mice were kindly provided by Prof. Sonnenberg (NKI, Amsterdam, the Netherlands) [28]. Glutaraldehyde fixation was used to preserve both keratin and actin structure. Briefly, this fixation consisted of a first incubation step in 0.3% glutaraldehyde + 0.25% Triton in cytoskeleton buffer (10 mM MES pH 6.1, 150 mM NaCl, 5 mM EGTA, 5 mM glucose, and 5 mM MgCl_2_) for 2 min. and a second step with 0.5% glutaraldehyde in the same buffer for 10 min. Subsequently, the sample was treated with freshly made 0.1% NaBH_4_ in PBS. After fixation, samples were extensively washed with PBS and blocked with 5% BSA for 40 minutes. Staining was performed with rabbit anti-keratin 14 polyclonal antibody (Covance) and Phalloidin conjugated to Alexa Fluor 488 fluorophores (Invitrogen). Samples were incubated with a goat anti-rabbit secondary antibody labeled with Alexa Fluor 647 fluorophores (Invitrogen) afterwards. All the steps were performed at room temperature. Control experiments were also performed where the Phalloidin was labelled with Alexa Fluor 647 and the goat anti-rabbit antibodies with Alexa Fluor 488 to rule out color-related artefacts.

#### Microscope

Samples were imaged on a Leica SR-GSD microscope (Leica Microsystems, Wetzlar, Germany) equipped with 488 nm/300 mW, 532 nm/500 mW and 647 nm/500 mW lasers and an EMCCD camera (Ixon DU-897, Andor). A 160x oil immersion objective was used. Coverslips were mounted in a holder (Chamlide CMB, Korea) with 500 *μ*L consisting of PBS, merceptoethylamine (MEA, 50 mM) and newly developed oxygen scavenging system consisting of Oxyrase (OXYRASE Inc, Mansfield, Ohio, U.S.A, 3%) and lactate (20%) in PBS. Details will be described elsewhere. Before imaging, a waiting time of 30 min. was observed to allow the sample to stabilize and avoid initial drift. Images were then taken in TIRF mode at 100 frames per second with image sizes of 180 × 180 or 400 × 400 pixels; the backprojected pixel size was 100 nm. For all datasets, images with 642 nm illumination were acquired first.

#### Localization analysis of experimental data

The acquired movies were processed by estimating fluorophores’ positions using a fast algorithm [29] on a Quadro 5000 GPU (NVIDIA). The method for finding candidate regions of interest for position estimation has been documented in the literature [30]. Localizations corresponding to the same activation event were subsequently combined by grouping spatially nearby localizations (i.e. less than three times the sum of the localizations’ precisions apart) in subsequent frames into single localization events. The center position of the grouped localizations was determined as the weighted average of the localizations with the inverse of the squared localization precisions as weights. Localizations were then filtered based on the number of signal photons per localization event and the PSF width. Subsequently, localizations were corrected for lateral stage drift using frame-by-frame cross-correlation, as documented elsewhere [31, 32]. All images in which the experimentally obtained localizations are visualized were obtained by rendering visualizations as Gaussian blobs with a kernel size equal to the estimated localization precision. Pixels whose values were in the highest 2% (5% for images of actin and keratin) of all non-zero pixels were clipped to obtain sufficient contrast for display, and subsequently all intensities were linearly stretched between 0 and 255.

#### Color channel registration

Localizations of the Alexa Fluor 647 fluorophore (red) channel were mapped onto the Alexa Fluor 488 fluorophore (green) channel using affine mapping. This mapping was estimated in a least squares estimation procedure with 8 different datasets of (in total 448) fluorescent beads visible in both color channels. Briefly, 100 nm TetraSpeck microspheres (T7284 blue green orange and dark red, Life Technologies) were diluted to a ratio of 1:100 and dried on ultraclean coverslip. The bead-dried coverslips were mounted on the microscope with 500 *μ*L of MQ water and imaged on 8 different fields of view where beads were well separated. The beads were localized using the same algorithm as above. The target registration error of this mapping procedure was determined to be 16 nm (by leaving one of the recordings at a time out when computing the mapping so that it can be independently used to assess the error) [33].

## Results

### Simulated datasets

**Fig 2:**
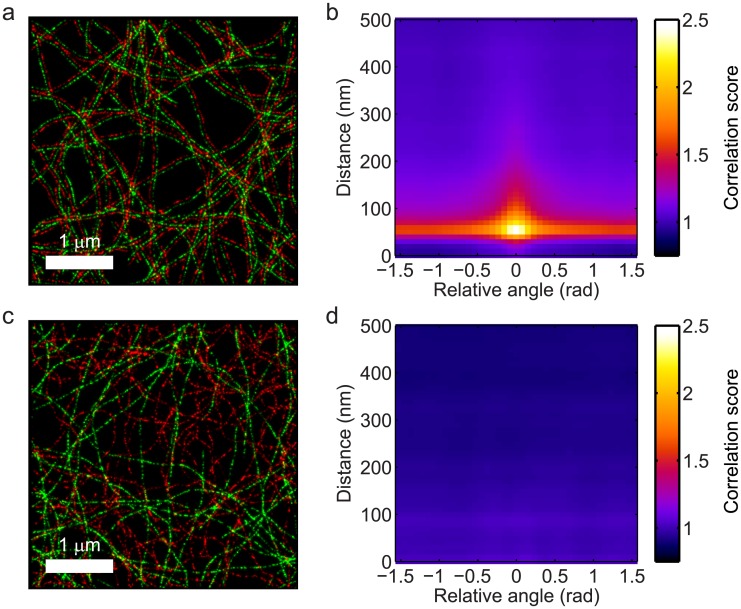
Co-orientation plot of parallel and unrelated filaments. (a) Simulated data of parallel filaments in two color channel channels and (b) the corresponding co-orientation plot, showing strong co-orientation at a distance of 50 nm between filaments. The co-orientation plot shows the cross-correlation between the color channels as a function of the distance between localizations in both channels (on the horizontal axis) and the difference in the orientations of the filaments those localizations belong to (on the vertical axis). (c) Simulated data of statistically independent filaments in two color channel channels and (d) the corresponding co-orientation plot, showing no substantial co-orientation.

To demonstrate the proposed co-orientation measurement method, we simulated two-color localization microscopy datasets of samples with filament networks in both channels with a well-defined relationship between them. As a first example, we used a sample with 200 filaments with a persistence length *ξ* = 5 *μ*m in the red color channel, labeled with 10^4^ fluorophores in total; each of these filaments was accompanied by a filament in the green color channel at a fixed distance of 50 nm. This resulted in the dataset shown in Fig 2a, and the corresponding co-orientation plot of the generalized cross-correlation function *c*(*r*, Δ*ϕ*) in Fig 2b (for a scale *s*_*o*_ = 200 nm for the orientation analysis). The plot shows the distance *r* between the localizations in both color channels on the vertical axis and the orientation difference *ϕ* between the filaments to which those localizations belong on the horizontal axis. The plot shows a clear peak at distance of approximately 50 nm and an orientation difference close to 0, confirming that filaments are accompanied by another filament at a distance of 50 nm in the other color channel. The enhanced correlation for larger angles *ϕ* is caused by the finite size of the orientation selective filters: when filaments cross or come in close proximity to each other, the filters give a non-zero response for orientations other than those of the filaments themselves. For larger distances *r* > 200 nm, *c*(*r*, *ϕ*) decays to a value of 1, meaning that filaments at those distances apart appear statistically independent from each other.

As a second in-silico example, we used a sample in which there was no relationship between the filaments in both color channels. Unlike the previous example, the filaments in the green channel were now independently generated, but with a persistence length *ξ* = 1 *μ*m. A representative example of a result under this condition (out of *n* = 500 simulations) is visualized in Fig 2c and the corresponding co-orientation plot in Fig 2d. Clearly, the values of *c*(*r*, *ϕ*) in Fig 2d are no longer substantially larger than 1, and there is no longer a noticeable dependence of the co-localization on the relative orientation of the filaments.

**Fig 3:**
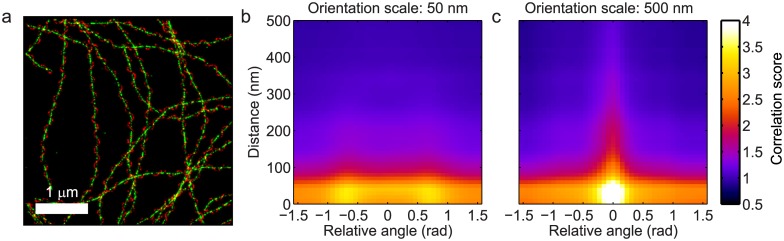
Orientation scale as a dimension for analysis. (a) Simulated data of filaments in the green color channel with filaments in the red channel twisted around them. (b) When the orientation is analyzed at a scale of 50 nm, the co-orientation plot shows two peaks at positive and negative angles between the filaments in both channels; (c) for a scale of 500 nm the peaks shift to the center of the plot indicating that the filaments in both channels appear to run in parallel at that scale. The smallest scale for the orientation analysis is determined by the FRC resolutions in both channels, are 34 nm (red) and 36 nm (green).

The third simulation example serves to illustrate the importance of the scale of the orientation analysis. For this example, 50 filaments labeled with 5,000 fluorophores were simulated for the red channel as before. The filaments in the red channel were twisted around the green filaments with a maximum separation of 50 nm and with a periodicity of one twist per 300 nm. The resulting dataset is visualized in Fig 3a. Co-orientation plots for these data were computed for scales *s*_*o*_ = 50 nm and *s*_*o*_ = 500 nm for the orientation analysis, which are shown in Fig 3b and 3c respectively. The plot for *s*_*o*_ = 50 nm shows two peaks at orientation differences of about ±40°, whereas the plot for *s*_*o*_ = 500 nm only has a single peak at ±0°. Thus these plots express how indeed the filaments in both channels display co-orientation at larger length scales, although at a shorter length scale there is a signature of the filaments crossing each other. This shows that the scale *s*_*o*_ of the orientation analysis can itself be used as a separate dimension for the analysis of co-orientation in an extensive co-orientation assay. The shortest length scale for which the orientation analysis could be meaningfully applied is determined by the resolution of the images [14]; at shorter length scales the data do not contain enough information about the filaments for an accurate analysis.

### Significance testing

**Fig 4:**
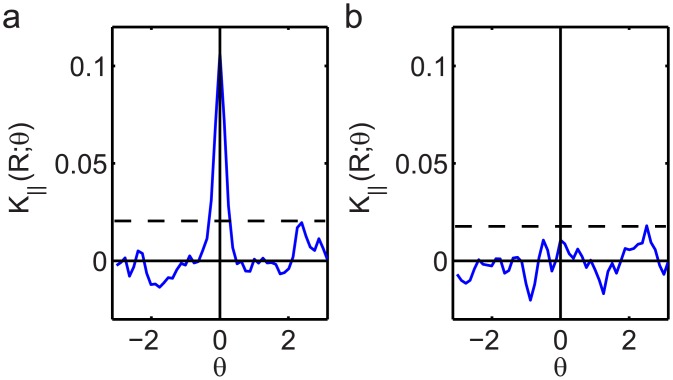
Statistical significance test results on simulated data. (a) The normalized anisotropic Ripley’s K statistic *K*_∥_(*R*) quantifies the co-orientation strength. Rotation over an angle *θ* of the color channels in Fig 2a relative to each other leads to a rapid decline of *K*_∥_(*R*); the residual fluctuations can be used to determine that the value *K*_∥_(*R*) at *θ* = 0 exceeds the threshold for statistical significance at the 0.01 level (dashed line). (b) The same plot for the data show in Fig 2c indicates that the co-orientation there is not significant for *θ* = 0.

The question that arises upon inspection of the co-orientation plots is for which values of *c*(*r*, Δ*ϕ*) the co-orientation can be said to be statistically significant. To this end we computed the normalized anisotropic Ripley’s K parameter *K*_∥_(*R*) with *R* = 200 nm for the simulated datasets in Fig 2 to quantify the co-orientation strength. Subsequently, we applied the significance test outlined in the materials and methods section, which extracts the uncertainty in *K*_∥_(*R*) by rotating the image in the green channel with respect to the red channel over 49 equally spaced angles *θ* between 0 and 2*π* and recomputing *K*_∥_(*R*) for every rotation. The profiles of *K*_∥_(*R*) as a function of the rotation angle *θ* for the datasets in Fig 2 are shown in Fig 4. The dashed line in the plot indicates the minimum value of *K*_∥_(*R*) at *θ* = 0 for which it would be significant at a significance level of 0.05. The value of *K*_∥_(*R*) for the parallel filaments in Fig 2a turned out to be statistically significant (*p* = 2.0 × 10^−37^ ≪ 10^−3^), whereas the value of *K*_∥_(*R*) for the unrelated filaments shown in Fig 2c was not (*p* = 0.079).

We validated the proposed significance test by simulating 500 datasets where the filaments in both color channels were independent in the same manner as for the data shown in Fig 2c. For each of these simulations we applied the proposed significance test and computed the p-value for the value of *K*_∥_(*R*) at *θ* = 0 for *R* = 200 nm. We found that the p-values returned by the test were consistent with a uniform distribution between 0 and 1 (see S4 Fig): a one-sample two-sided Kolmogorov-Smirnov test revealed no significant difference at a 0.05 significance level (*p* = 0.47). This is exactly what is required, as the returned p-values should report the probability of obtaining values of *K*_∥_(*R*) larger than the one being tested if the null hypothesis is true. Additionally, the assumption that *K*_∥_(*R*) is normally distributed was not rejected in a Shapiro-Wilks test at a significance level 0.05 (*p* = 0.42). However, 38 of 500 the simulated datasets had a p-value smaller than 0.05, which is significantly more than the expected 25, indicating that the p-values obtained from the proposed significance test are not exact. This is attributed to the RMS error of 31% in the estimated standard deviation of *K*_∥_(*R*), since the normality of *K*_∥_(*R*) itself was not rejected. The test can still be used though, provided that a somewhat more conservative threshold than 0.05 is chosen for the p-value.

### Application to experimental data of cytoskeletal filaments

**Fig 5:**
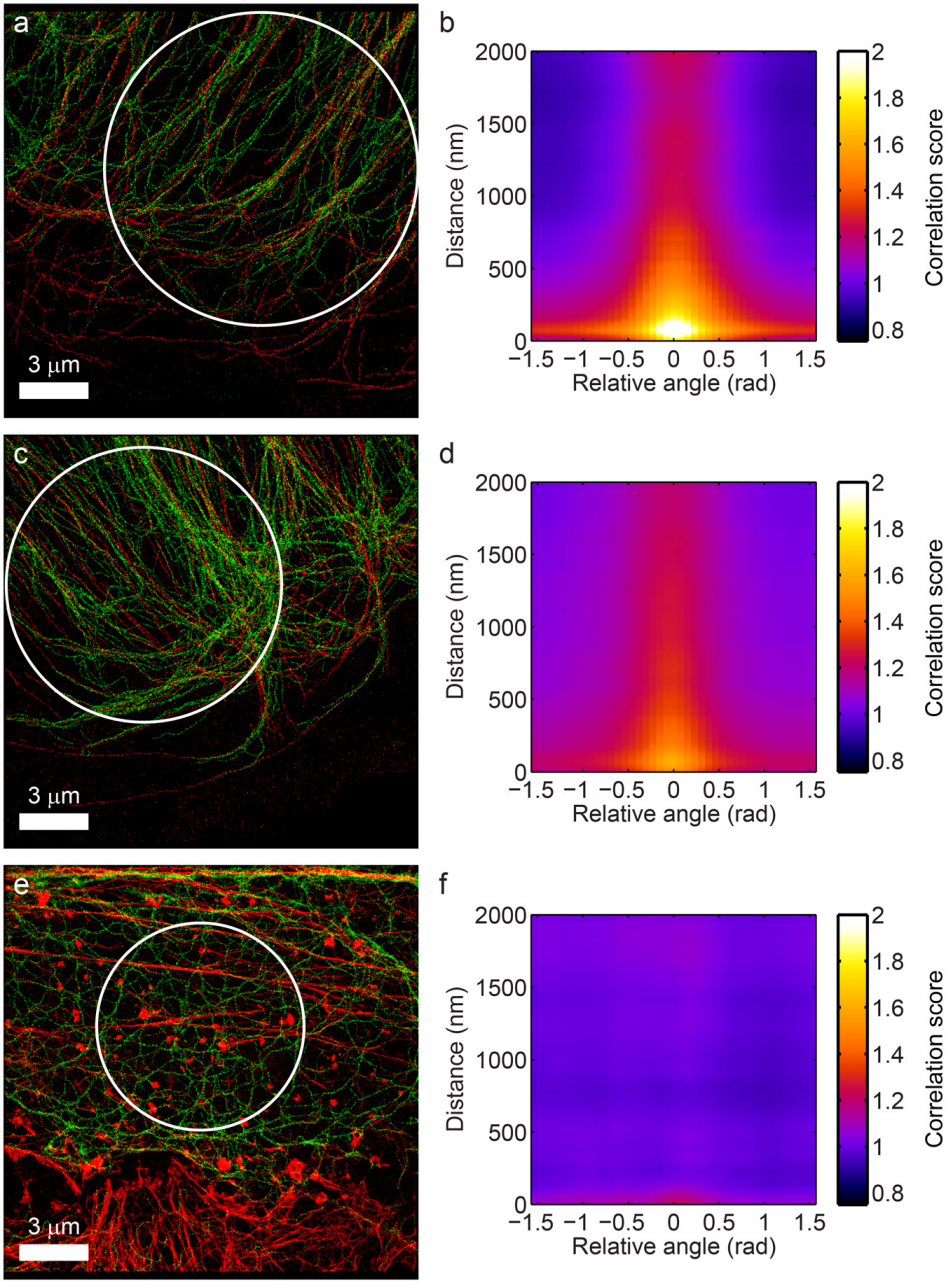
Co-orientation analysis for experimental data of tubulin and vimentin and of actin and keratin. (a) and (c) Localization microscopy images of tubulin (red) and vimentin (green) at stable cell edges. The co-orientation plots for the ROIs demarcated by the white circles are shown in (b) and (d), showing clear co-orientation at distances up to 500 nm (with a scale *s*_*o*_ = 200 nm for the orientation analysis; results for (a) for multiple different scales *s*_*o*_ are shown in S5 Fig). (e) Localization microscopy image of actin (red) and keratin (green). The co-orientation plot in (f) for the selected region of interest shows no significant co-orientation.

We applied the co-orientation analysis to experimental data of tubulin and vimentin and of actin and keratin. Multicolor localization microscopy images of tubulin and vimentin were obtained from primary human umbilical vein endothelial cells. Fig 5a and 5c show two clear example results at stable cell edges, with tubulin in red and vimentin in green. The corresponding co-orientation plots in Fig 5b and 5d confirm the strong co-orientation effect that appears to be present. The effect appears stronger in b than in d, due to the lower density of the filaments which leads to a stronger apparent bundling of the filaments. Correspondingly, the co-orientation strength parameter *K*_∥_(*R*) for the selected circular ROI in Fig 5a is larger than that in the ROI in Fig 5c, which are respectively 0.22 and 0.12 for *R* = 500 nm; in both ROIs the co-orientation is statistically significant (*p* ≪ 10^−3^). The value of *R* = 500 nm was chosen here such that the *K*_∥_(*R*) just incorporates the primary peak in the co-orientation plots in the analysis. The observed co-orientation could also just be seen when the co-orientation analysis was applied to the TIRF images of the cells shown in Fig 5a and 5c (see S7 Fig). Generally though, the higher resolution of SR microscopy is much more suitable, and often will be necessary, to detect the co-orientation between these intricate filament networks. Note that the filament networks in these images show a clear preferential direction in these cells. Local deviations from these global trends could be investigated for example by filtering out the dominant filament orientations in the orientation space representations of the tubulin and vimentin images. Alternatively, the co-orientation plot could be normalized with respect to its average value at each distance *r* in order to determine how the alignment changes with *r* independent of the co-localization.

The observed co-orientation between vimentin and tubulin is not a universal feature of any image showing two types of filaments. Consider for example Fig 5e, which shows a localization microscopy image of actin (green) and keratin (red) obtained from plectin deficient keratinocytes. As opposed to the previous images of tubulin and vimentin, there is no apparent co-orientation between actin and keratin: the corresponding co-orientation plot in Fig 5f does not exhibit a strongly peaked correlation score for small distances and small relative angles between the actin and keratin filaments. Indeed, no significant co-orientation (*p* = 0.20) was found in a statistical significance test for *R* = 500 nm (*p* = 0.065 for *R* = 200 nm).

**Fig 6:**
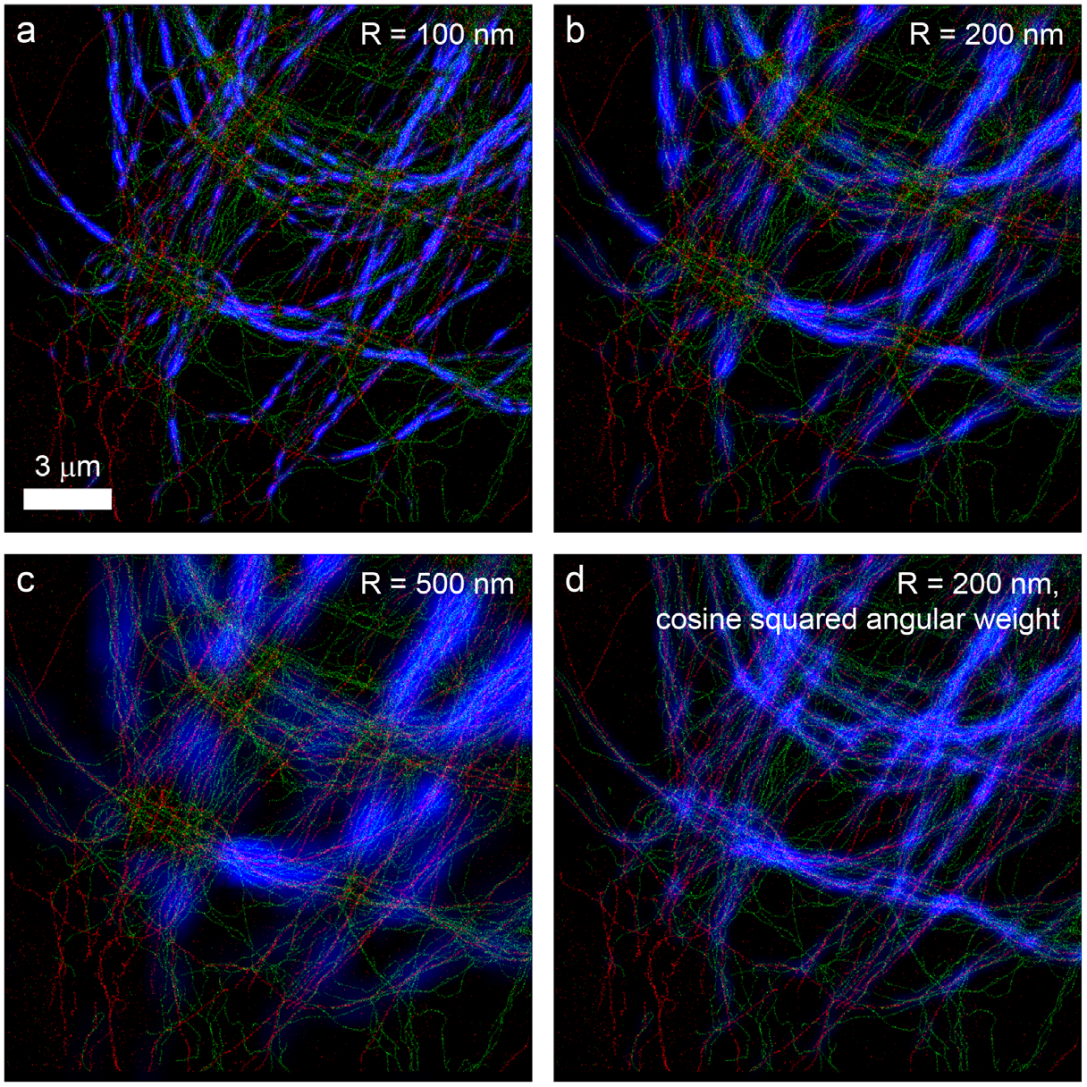
Visualization of the local co-orientation strength. (a-c) Localization microscopy images of tubulin (red) and vimentin (green). Blue overlays show the local co-orientation strength *K*_∥_(*R*) in order to highlight the regions with the strongest local co-orientation. Increasing *R* causes more filaments that are further apart from each other to contribute to *K*_∥_(*R*), but also causes *K*_∥_(*R*) to appear less localized. (d) The same image as (b), but with the cos(2*ϕ*) weight in the computation of *K*_∥_(*R*) in Eq 7 replaced by a cos^2^(*ϕ*) weight. This provides a visualization in which crossing filaments do not cancel the contributions to the local co-orientation strength of parallel filaments. However, this visualization is also sensitive to regions with mere co-localization where filaments are not aligned.

To visualize how the co-orientation between filaments varies across the image, we evaluated the local co-orientation strength *K*_∥_(*R*) in overlapping subregions of the image. The resulting values are then shown as an overlay in the blue color channel on top of the image of the filaments. Fig 6 shows an example of tubulin and vimentin filaments with this overlay for different values of *R*, with subregion sizes equal to 3*R*. The blue overlay effectively highlights regions with the strongest local co-orientation, where high densities of filaments with similar orientations are within a distance *R* from each other. Increasing *R* causes more filaments to positively contribute to *K*_∥_(*R*). However, it also leads to a less localized evaluation of the co-orientation strength. Regions in the image with crossing filaments exhibit lower values, because locally there is evidence both for and against orientational alignment of the tublin and vimentin. An alternative visualization method that does not give this low response with crossing filaments is demonstrated in Fig 6d. In this method the cos(2*ϕ*) weight in the computation of *K*_∥_(*R*) in Eq 7 is replaced by a cos^2^(*ϕ*) weight. This leads to more connected regions with high values in the blue channel, but this visualization also highlights regions with mere co-localization where filaments are not aligned.

**Fig 7:**
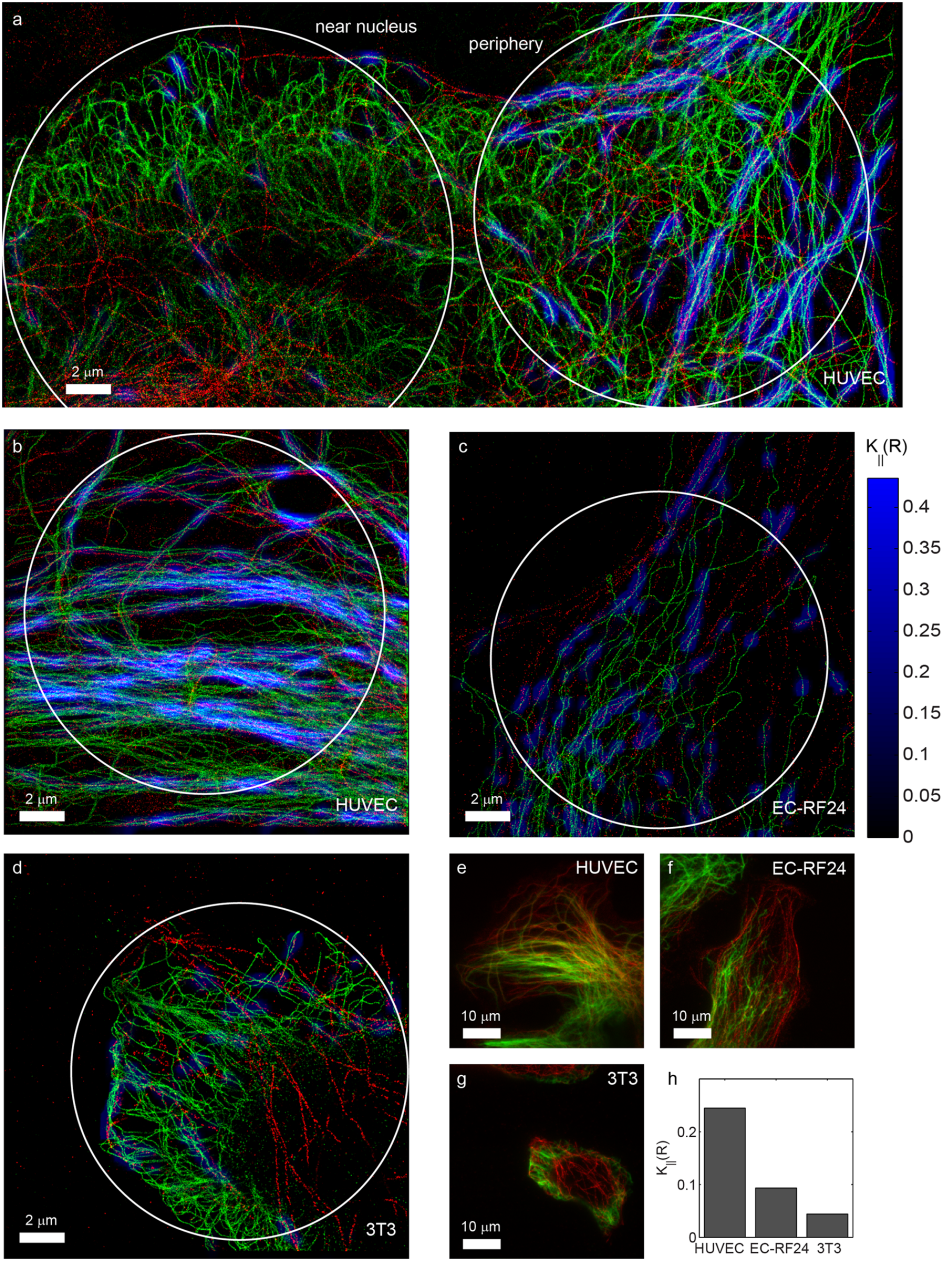
Co-orientation strength in endothelial and fibroblast cells. Localization microscopy images of tubulin (red) and vimentin (green) in various cell types. (a) Large SR image of a HUVEC cell, showing that co-orientation is predominantly observed in the peripheral parts (right), and not near the nucleus (left). (b-d) Higher magnifications of comparable peripheral parts of (b) a HUVEC cell showing extensive co-orientation, (c) a EC-RF24 endothelial cell with less, but still significant co-orientation, and (d) a NIH-3T3 fibroblast as an example of a cell-type with very little co-orientation. (e-g) TIRF images corresponding to b, c and d and (h) quantification of the co-orientation strength for the circular ROIs in these three examples for *R* = 200 nm.

In larger images (i.e. of 18 × 40 *μ*m), it was apparent that co-orientation between vimentin and tubulin occurred predominantly in the periphery of the cells, whereas at the center, close to the nucleus, co-orientation appeared substantially less. When we compared the right and left half of Fig 7a respectively, we found *K*_∥_(*R*) = 0.11 (*p* ≪ 10^−3^) and *K*_∥_(*R*) = 2.9 × 10^−2^ (*p* ≪ 10^−3^) respectively for *R* = 200 nm.

We next investigated whether co-orientation between tubulin and vimentin is a generic property of these filaments. We therefore compared data from HUVEC cells (Fig 7b) to data obtained from NIH-3T3 fibroblasts (Fig 7d), which also express both filament systems. Remarkably, little if any co-orientation was observed throughout the cell in these fibroblasts: for the ROI in Fig 7d we found no statistically significant co-orientation (*K*_∥_(*R*) = 4.4 × 10^−2^ and *p* = 0.14 for *R* = 200 nm). We also did not observe a difference between peripheral and more central parts of the cells. This may reflect lineage-dependency, i.e. a difference between endothelial cells and fibroblasts. We therefore also studied a cultured endothelial cell line, EC-RF24 (Fig 7c). Indeed, we observed significant co-orientation (*K*_∥_(*R*) = 9.4 × 10^−2^ and *p* ≪ 10^−3^ for *R* = 200 nm), but both strength and extent of colocalization appeared less than in HUVEC cell (*K*_∥_(*R*) = 0.24 and *p* ≪ 10^−3^ for *R* = 200 nm).

These results show that our analysis methods makes it possible to quantitatively address biological co-orientation. Associations between different filament systems have recently attracted significant attention and may either indicate the existence of physical crosslinks between the filaments [34] or, perhaps, reflect deposition of intermediate filaments following their transport along microtubuli [35]. Our analysis tools will enable addressing such questions in an unbiased and quantitative manner.

## Discussion

In this work, we describe a framework for the quantitative analysis of co-orientation: the simultaneous co-localization and orientational alignment of structures in images. In this framework we consider generalized cross-correlation between color channels as a function of spatial separation and orientational difference of structures. Additionally we quantify the (local) co-orientation strength using an anisotropic Ripley’s K parameter and use it to test the statistical significance of the co-orientation. Our co-orientation analysis sensitively and quantitatively describes spatial association between vimentin and microtubuli in HUVEC cells. Moreover, this association is cell-type specific and appears to occur predominantly in the cell periphery.

Although the results presented in this manuscript are obtained using simulated and experimental localization microscopy datasets, the methods proposed here can be analogously applied to data obtained with other superresolution microscopy techniques as well as widefield and confocal microscopy data if the resolving power is appropriate for distinguishing the structures (e.g. filaments) in those images.

The co-orientation measurement is affected to some extent by experimental factors such as autofluorescence and background fluorescence from out-of-focus structures, apparent blurring of structures by the imaging system (e.g. due to diffraction or localization error), cross-talk between color channels, noise, and stochasticity in the fluorescent labeling (see S1 Text for a detailed discussion). Particularly the localization error in localization microscopy and analogously the point-spread function in other microscopy techniques may have substantial effects on the measurement outcomes. Firstly, they will lead to a change in the effective scale at which the orientation of filaments is assessed. Secondly, they smear out the generalized cross-correlation function $c\left(\Delta \vec{x},\Delta \phi \right)$, causing the peaks in the co-orientation plot to decrease in magnitude and shift to larger values of the distance between filaments.

There are several practical aspects that merit attention when interpreting the outcome of the orientation measurement and significance test. Firstly, it is important to note that the measured co-orientation strength *K*_∥_(*R*) may decrease if the density of co-oriented filaments in the field of view increases. This merits attention when comparing the measurement outcomes for different cells or cell lines if their filament densities are not similar. The co-orientation measurement could be made less sensitive by changing the average values per channel in the denominator of $c\left(\Delta \vec{x},\Delta \phi \right)$ into the root-mean-square values; however, this normalization has the important disadvantage of being sensitive to changes in noise levels, density of fluorescent labels on the filaments, or localization precision.

Secondly, the density of filaments also affects the validity of the significance testing method. Its derivation assumes a Gaussian distribution of *K*_∥_(*R*) under the null hypothesis, which may not hold if the number of filaments in the field of view is small. Furthermore, the accuracy with which the standard deviation of *K*_∥_(*R*) is estimated under the null hypothesis also depends on the number of filaments in the field of view. Therefore it is recommended to consider a more conservative significance level than 0.05 when testing for statistical significance. Also, care should be taken with strong co-localization in the absence of co-orientation, as it violates the assumption of rotation invariance under the null hypothesis that is built into the test.

Thirdly, if no statistically significant co-orientation is detected, this does not imply that no co-orientation effect is present. The likelihood of successfully detecting co-orientation depends on how different the co-orientation effect appears from random variations in the proximity and alignment of unrelated filaments. Stronger co-localization or alignment therefore increase the detection probability. In addition, the detection probability will be higher for larger numbers of filaments as random variations tend to average out more. Of course, imaging more samples will increase the probability of detection as well, provided that a suitable procedure for simultaneously performing multiple significance tests is used (e.g. false discovery rate control).

The visualization schemes that were proposed either underemphasize co-orientation in regions with crossing filaments or overemphasize regions where co-localization with little orientational alignment is present. These visualization schemes may be improved in several ways. Firstly, a method for detecting regions with crossing filaments in both color channels could identify where each scheme is most appropriate. This could be achieved by a crossing detector per color channel and then feeding the output into a co-localization measure. Secondly, higher order terms in the Fourier series expansion of $c\left(\Delta \vec{x},\Delta \phi \right)$ could be used to describe the local geometry in regions with crossing filaments. For example, the term with cos(4*ϕ*) rather than cos(2*ϕ*) expresses co-orientation between a filament in one channel and one of two orthogonal filaments in the other channel.

Finally, the quantitative approach presented in this manuscript was specifically focused on the analysis of co-orientation, i.e. the combination of co-localization of filaments and the alignment in their orientations. However, the quantitative framework presented here can be applied more generally to the analysis of co-localization in conjunction with other geometric properties, such as the curvature or length of filaments or diameter of filament bundles. The analysis would then entail the computation of the cross-correlation between color channels as a function of these geometric properties, possibly at multiple measurement scales. Deriving a scalar metric for the magnitude of the observed effect similar to *K*_∥_(*R*) then allows for the assessment of the local effect size and testing of its statistical significance. Approaches such as these will be of great use for exploiting the wealth of information provided by superresolution microscopy images for studying the spatial arrangements of cytoskeletal filaments and associated proteins relative to each other.

## Supporting Information

S1 FigThe effect of filament separation and field of view size on co-orientation.Simulated datasets consisting of two parallel straight lines with a density of fluorophores of one per 8 nm. The datasets for (a) and (c) differ in the distance between the filaments, which is 50 nm and 200 nm respectively. (b) and (d) show that this causes a shift and decrease in the peak of the co-orientation plot. The decrease is due to the larger radius over which $c\left(\Delta \vec{x},\Delta \phi \right)$ is averaged; *K*_∥_(*R*) for *R* > 200 nm would not be similarly affected. The datasets for (c) and (e) differ in the size of the field of view, resulting in an increase in the peak from the plot in (d) to the plot in (f).(TIF)

S2 Fig The effect of non-co-localizing filaments on co-orientation. The two lines to the right in (a) are obtained in the same manner as the two lines in S1a Fig. The additional line on the left in the green channel causes a decrease in the co-orientation (compare (b) with S1b Fig).
(EPS)

S3 Fig The effect of filament density on co-orientation. (a) Parallel lines with the same density of localizations and total length of the filaments as in S1a Fig; the resulting co-orientation plot in (b) is not substantially different than in S1b Fig. Doubling the total filament length from (a) to (c) and (e) does affect the co-orientation plot (compare (b) with (d) and (f)). The co-orientation is not affected by the shift of the left red filament from the left green filament in (c) to the right green filament in (e) (compare (d) and (f)).
(EPS)

S4 Fig Validation of the significance test. Results are obtained for 500 simulated datasets generated in the same manner as Fig 2c. Application of the significance test results in a uniform distribution of P-values, as evidenced by the histogram in (a) and empirical cumulative distribution function in (b). The values of *K*_∥_(*R*) exhibit a Gaussian distribution in the histogram in (c) and the quantile-quantile plot in (d).
(EPS)

S5 Fig Influence of orientation scale in experimental data. (a-e) Co-orientation plots for the data shown in Fig 5a at various values of the scale of the orientation analysis *s*_*o*_. Clearly, the filaments appear more aligned and the correlation in the orientation persists over longer distances with increasing *s*_*o*_. At the smallest scale *s*_*o*_ = 100 nm the low signal-to-noise-ratio of the images leads to a strong reduction of the correlation. (f-j) Images of the corresponding orientation selective filter kernels (scalebar: 1 *μ*m).
(EPS)

S6 Fig Saturation of the local co-orientation overlay. (a-f) The same image as Fig 6b for various fractions of the brightest pixels that are clipped for the visualization (default is 3%). Clearly, many more filaments have a bright overlay in blue for higher fractions of clipped pixels due to the fairly large range of the local values of *K*_∥_(*R*). However, this comes at the cost of the contrast in the blue channel among different regions where the local co-orientation is strong.
(TIF)

S7 Fig Co-orientation analysis for TIRF images of tubulin and vimentin. (a-d) TIRF images of the same samples as shown in Fig 5a and 5c were used for co-orientation analysis. The co-orientation plots in (b) and (d) shows that the analysis can be applied to these TIRF images and does reveal the co-orientation between tubulin and vimentin (with a scale *s*_*o*_ = 200 nm for the orientation analysis). However, the correlation scores are much lower due to the blurring effect of the point spread function (see S1 Text for a more detailed analysis). The higher resolution of localization microscopy is therefore much more suitable, and often will be necessary, to detect the co-orientation between these intricate filament networks.
(EPS)

S1 Text Supporting theoretical analyses. First a theoretical derivation of the equations used in the significance testing methods is provided. Secondly an analysis is presented of the impact of various experimental factors on the accuracy of the co-orientation measurement.
(PDF)
