# Supplementary figures and images for: Co-Orientation: Quantifying Simultaneous Co-Localization and Orientational Alignment of Filaments in Light Microscopy
Source: PLoS One. 2015 Jul 10;10(7):e0131756. doi: 10.1371/journal.pone.0131756 (PMC4498647; doi:10.1371/journal.pone.0131756)

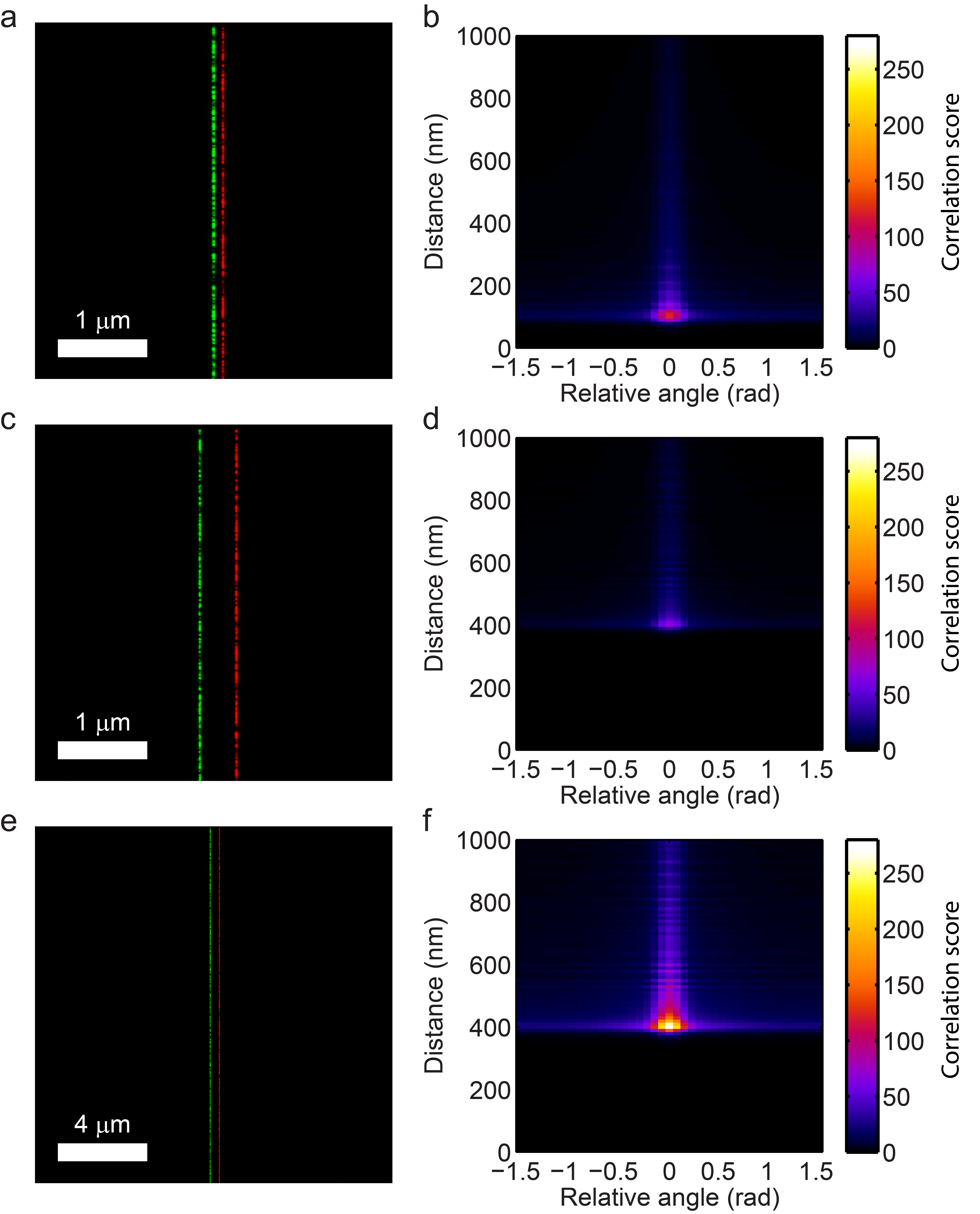

Supplement: S1 Fig — Simulated datasets consisting of two parallel straight lines with a density of fluorophores of one per 8 nm. The datasets for (a) and (c) differ in the distance between the filaments, which is 50 nm and 200 nm respectively. (b) and (d) show that this causes a shift and decrease in the peak of the co-orientation plot. The decrease is due to the larger radius over which c(Δx→,Δϕ) is averaged; K ∥(R) for R > 200 nm would not be similarly affected. The datasets for (c) and (e) differ in the size of the field of view, resulting in an increase in the peak from the plot in (d) to the plot in (f). (TIF) [file pone.0131756.s001.tif]

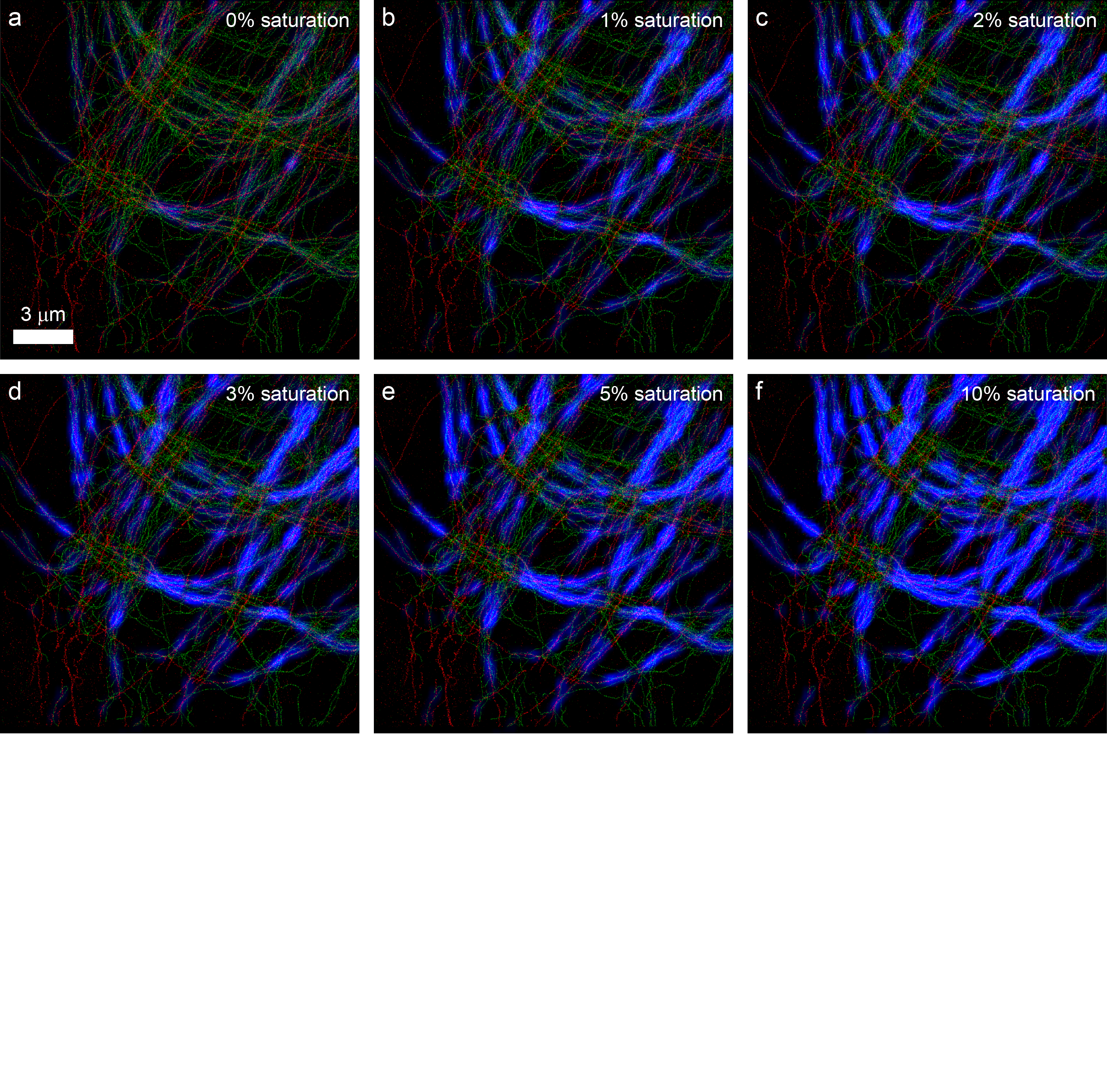

Supplement: S6 Fig — (a-f) The same image as Fig 6b for various fractions of the brightest pixels that are clipped for the visualization (default is 3%). Clearly, many more filaments have a bright overlay in blue for higher fractions of clipped pixels due to the fairly large range of the local values of K ∥(R). However, this comes at the cost of the contrast in the blue channel among different regions where the local co-orientation is strong. (TIF) [file pone.0131756.s006.tif]
