# Supplementary material for: Co-Orientation: Quantifying Simultaneous Co-Localization and Orientational Alignment of Filaments in Light Microscopy
Source: PLoS One. 2015 Jul 10;10(7):e0131756. doi: 10.1371/journal.pone.0131756 (PMC4498647; doi:10.1371/journal.pone.0131756)
Supplement: S1 Text — First a theoretical derivation of the equations used in the significance testing methods is provided. Secondly an analysis is presented of the impact of various experimental factors on the accuracy of the co-orientation measurement. (PDF) [file pone.0131756.s008.pdf]

S1 Text: Supporting theoretical analyses

Derivation of the proposed significance test

In this section we provide a justification for the significance testing procedure described in the main text. The first assumption that is made is that $K_{||}(R)$ follows a Gaussian distribution. This is an hypothesis based on the idea that the images I_1 and I_2 are actually sums of images of filaments. These filaments therefore all contribute to $K_{||}(R)$ in an additive manner, if we assume that the average density per channel is more or less fixed. Assuming then that the contribution of each filament has a well-defined mean and variance and that positions and orientations of filaments that are far apart are only weakly correlated, we can invoke the central limit theorem which describes that the distribution of $K_{||}(R)$ should tend towards a Gaussian distribution.

The task is then to estimate the variance of $K_{||}(R)$, assuming that the null hypothesis that the filamens in both color channels are unrelated is true. This assumption can be used because the significance test determines the probability under the null hypothesis of finding an outcome for $K_{||}(R)$ that is at least as extreme as the current value. Now suppose that we have measured $K_{||}(R;\theta)$ for several equally spaced angles θ between 0 and 2π. We know that under the null hypothesis, the expected value for $K_{||}(R)$ is 0.

The variance of $K_{||}(R;\theta=0)$ can be expressed as:

$$\mathrm{Var}\left(K_{||}(R;\theta=0)\right)=\frac{1}{n_\theta}\sum_\theta \mathrm{Var}\left(K_{||}(R;\theta)\right)=\frac{1}{n_\theta}\sum_\theta\left\langle K_{||}(R;\theta)^2\right\rangle, \tag{1}$$

since rotations of one color channel by an angle θ should not affect any expectation values under the null hypothesis: rotating one of the channels still leaves two independent images. Note that the notation $\langle . \rangle$ denotes the expected value here instead of the averaging operation. The discrete angle Fourier transform of $K_{||}(R;\theta)$ is defined as:

$$\hat{K}_{||}(R;q_\theta)\equiv\sum_\theta K_{||}(R;\theta)\exp\left(-iq_\theta\theta\right) \tag{2}$$

By applying Parseval's theorem, we find that:

$$\left\langle K_{||}(R;\theta=0)^2\right\rangle=\left\langle\frac{1}{n_\theta^2}\hat{K}_{||}(R;q_\theta=0)^2\right\rangle+\left\langle\frac{1}{n_\theta^2}\sum_{q_\theta\neq 0}\mathrm{Re}\left(\hat{K}_{||}(R;q_\theta)\right)^2+\mathrm{Im}\left(\hat{K}_{||}(R;q_\theta)\right)^2\right\rangle, \tag{3}$$

where Re(.) and Im(.) denote the real and imaginary part of a complex number respectively. By definition we have that,

$$\frac{1}{n_\theta}\hat{K}_{||}(R;q_\theta=0)=\frac{1}{n_\theta}\sum_\theta K_{||}(R;\theta). \tag{4}$$

The next step now to realize that because of the invariance of the statistics with respect to rotation of one channel by an angle $\Delta\theta$, we find that the distribution of $K_{||}(R;\theta)$ is the same as that of $K_{||}(R;\theta+\Delta\theta)$. This in turn implies that also $\hat{K}_{||}(R;q_\theta)$ and $\hat{K}_{||}(R;q_\theta)\exp\left(-i\Delta\theta\right)$ are identically distributed, and by

extension also that $\mathrm{Re}\left(\hat{K}_{\parallel}\left(R;q_\theta\right)\right)$ and $\mathrm{Im}\left(\hat{K}_{\parallel}\left(R;q_\theta\right)\right)$ are identically distributed. Therefore:

$$\begin{aligned}\left\langle K_{\parallel}\left(R;\theta=0\right)^2\right\rangle &= \left\langle\left(\frac{1}{n_\theta}\sum_\theta K_{\parallel}\left(R;\theta\right)\right)^2\right\rangle+\left\langle\frac{2}{n_\theta^2}\sum_{q_\theta\neq 0}\mathrm{Im}\left(\hat{K}_{\parallel}\left(R;q_\theta\right)\right)^2\right\rangle && (5)\\ &= \left\langle\left(\frac{1}{n_\theta}\sum_\theta K_{\parallel}\left(R;\theta\right)-1\right)^2+\frac{1}{2n_\theta}\sum_\theta\left(\frac{1}{2}K_{\parallel}\left(R;\theta\right)-\frac{1}{2}K_{\parallel}\left(R;-\theta\right)\right)^2\right\rangle. && (6)\end{aligned}$$

This shows that σ_K^2 is an unbiased estimator of the variance of $K_{\parallel}\left(R;\theta=0\right)$ if it is defined as:

$$\sigma_K^2=\left(\frac{1}{n_\theta}\sum_\theta\left(K_{\parallel}\left(R;\theta\right)-1\right)\right)^2+\frac{1}{2n_\theta}\sum_\theta\left(K_{\parallel}\left(R;\theta\right)-K_{\parallel}\left(R;-\theta\right)\right)^2. \tag{7}$$

The rationale for looking only at the imaginary part of $\hat{K}_{\parallel}\left(R;q_\theta\right)$ is that true co-orientation effects are expected to be symmetric with respect to positive and negative rotation angles θ. Therefore, if the null hypothesis is false, this estimate of the variance will be lower and thus make rejection of the null hypothesis using this test more likely.

Influence of experimental factors on the co-orientation measurement

The following sections provide a brief discussion of the most important experimental factors that affect the outcome of the co-orientation measurement. To make the discussion applicable, we will discuss both the effects that play a role in localization microscopy, as well as the analogous effects in microscopy methods that do not rely on stochastically activated or switching fluorophores. The latter will be referred to as deterministic microscopy techniques, and include among others widefield microscopy, confocal microscopy, stimulated emission depletion (STED) [1,2], and structured illumination microscopy (SIM) [3,4].

Background Background intensities in deterministic microscopy tend to result from out of focus structures and therefore typically do not possess much fine detail. Similarly, in localization microscopy the localizations due to this background do not show much small scale variation. Consequently, the effect of these on the orientation measurement will be limited as low spatial frequencies associated with large scale variations are suppressed by the orientation selective filters $\hat{\Phi}\left(\vec{q};\phi\right)$.

The primary effect of the background then is to increase the average values of the images I_1 and I_2. If we assume that the background is uncorrelated to the signal intensities I_1 and I_2 and we denote the background intensities in channels 1 and 2 with b_1 and b_2 respectively, then we find that the result is that:

$$c\left(\Delta\vec{x},\Delta\phi\right)\to 1+\left(c\left(\Delta\vec{x},\Delta\phi\right)-1\right)\left(\frac{\langle I_1\rangle}{\langle b_1\rangle+\langle I_1\rangle}\right)\left(\frac{\langle I_2\rangle}{\langle b_2\rangle+\langle I_2\rangle}\right) \tag{8}$$

Point spread function and localization error The localization error, and analogously also the point spread function for deterministic microscopy, has a double effect when it comes to orientation analysis.

Firstly, the effective scale at which the orientation is analyzed changes. Suppose for a moment that the image I corresponds to an object ψ. If the point spread function $h\left(\vec{x}\right)$ is approximated with a two-dimensional Gaussian function with standard deviation σ, then the expected Fourier transform of the image I can be written as:

$$\left\langle\hat{I}\left(\vec{q}\right)\right\rangle=\hat{\psi}\left(\vec{q}\right)\hat{h}\left(\vec{q}\right)=\hat{\psi}\left(\vec{q}\right)\exp\left(-2\pi^2\sigma^2q^2\right) \tag{9}$$

Here $\hat{\psi}(\vec{q})$ and $\hat{h}(\vec{q})$ are the Fourier transforms of $\psi(\vec{x})$ and $h(\vec{x})$ respectively. A similar relationship holds for the expected Fourier spectrum in localization microscopy if all localizations are obtained with have the same localization precision σ [5]. The expected outcome of the application of the filter $\hat{\Phi}(\vec{q};\phi)$ to I is therefore equivalent to:

$$\left\langle \hat{I}(\vec{q})\,\hat{\Phi}(\vec{q};\phi)\right\rangle = \hat{\psi}(\vec{q})\exp\left(-2\pi^2\sigma^2q^2\right)\hat{\Phi}(\vec{q};\phi) \tag{10}$$

It can be shown that up to a multiplicative constant, $\exp\left(-2\pi^2\sigma^2q^2\right)\hat{\Phi}(\vec{q};\phi)$ can be described by the same expression as $\hat{\Phi}(\vec{q};\phi)$ if we make the following parameter substitution:

$$s_o \to s_o\sqrt{1+4\pi^2\sigma^2w_q^2} \tag{11}$$

$$w_q \to \frac{w_q}{\sqrt{1+4\pi^2\sigma^2w_q^2}} \tag{12}$$

Since $w_q s_o$ is typically chosen to be a fixed fraction, it follows that the net result is that nonzero σ increases the scale s_o at which the orientation is evaluated. In theory, the equations above also provide a means of correcting for this effect of increased scale.

The second consequence of the point spread function or localization error is the blurring of $c(\Delta\vec{x},\Delta\phi)$. If $\hat{h}(q=0)=1$, then the average image value $\langle I_l\rangle_{\vec{x}}$ remains unaffected. However, the numerator in the expression for $c(\Delta\vec{x},\Delta\phi)$ can be seen as a convolution between $\tilde{I}_1(\vec{x},\phi)$ and $\tilde{I}_2(-\vec{x},-\phi)$ over both $\vec{x}$ and ϕ. The effect of convolving these with the point spread functions is therefore:

$$c(\vec{x},\phi) \;\to\; c(\vec{x},\phi) * (h_1(\vec{x})\,\delta(\phi)) * (h_2(-\vec{x})\,\delta(-\phi)) \tag{13}$$

$$= c(\vec{x},\phi) * (h_1(\vec{x}) * h_2(-\vec{x}))\,\delta(\phi)\ , \tag{14}$$

where $*$ denotes the convolution operator and $\delta(\phi)$ is the Dirac delta function. Note that if $h_1(\vec{x})$ and $h_2(-\vec{x})$ are known, then it might be possible to correct for their effects using deconvolution methods based on this insight.

The significance of these effects depends on the ratio between s_o and σ. For localization microscopy σ can be quite small, on the order of 5 to 10 nm, which means that the the localization error should not affect the co-orientation measurement very substantially. For deterministic microscopy techniques, these effects will often be much more significant, because the PSF is typically much wider.

Channel cross-talk Channel cross-talk occurs when part of the intensity or localizations from molecules belonging in one color channel appears in the other channel. Typically, this fraction is quite homogeneous over the field of view, as the level of cross-talk is determined by the emission filters. If we neglect the influence of noise for now, then the intensity in channel l can be represented as:

$$I_l(\vec{x}) \to (1-f_{l,m})\,I_l(\vec{x}) + f_{m,l}I_m(\vec{x}) \tag{15}$$

where $f_{l,m}$ is the fraction of the intensity or localizations from molecules that are imaged with channel l ending up in channel $m \neq l$. Assuming for convenience that the minimal values of $I_l(\vec{x},\phi)$ and $I_m(\vec{x},\phi)$ are negligible, we find that:

$$\tilde{I}_l(\vec{x},\phi) \to \left(\frac{|(1-f_{l,m})\,I_l(\vec{x},\phi)+f_{m,l}I_m(\vec{x},\phi)|}{\int_{-\pi/2}^{\pi/2}|(1-f_{l,m})\,I_l(\vec{x},\phi')+f_{m,l}I_m(\vec{x},\phi')|\,d\phi'}\right)((1-f_{l,m})\,I_l(\vec{x})+f_{m,l}I_m(\vec{x}))\ . \tag{16}$$

This shows that the impact of channel cross-talk on $\tilde{I}_l(\vec{x},\phi)$ is nonlinear, due to the nonlinearity in the operation of taking the absolute value. Therefore it is difficult to predict the impact of cross-talk on the

co-orientation measurement. However, if the overlap of $I_1(\vec{x})$ and $I_2(\vec{x})$ after convolution with the filters $\Phi(\vec{x},\phi)$ is small, then the cross-talk will approximately result in:

$$\tilde{I}_l(\vec{x},\phi) \rightarrow (1 - f_{l,m})\,\tilde{I}_l(\vec{x},\phi) + f_{m,l}\tilde{I}_m(\vec{x},\phi)\ . \tag{17}$$

This implies that $c(\Delta\vec{x},\Delta\phi)$ will be corrupted by contributions due to the autocorrelation of $\tilde{I}_1(\vec{x},\phi)$ and $\tilde{I}_2(\vec{x},\phi)$ with respect to both $\vec{x}$ and ϕ.

In practice, effects of channel cross-talk should not be very substantial given that many fluorophores are now available for large parts of the visible spectrum.

Noise Noise sources will usually not affect the expected value of $c(\Delta\vec{x},\Delta\phi)$ for deterministic microscopy techniques, provided that they are uncorrelated between color channels and that they do not affect the expect value of I_1 and I_2 These are reasonable assumptions for most types of high resolution microscopy, where dominant noise sources are usually photon counting shot noise and additive readout noise.

For localization microscopy, the stochasticity in the number of acquired localizations per fluorescent emitter plays a similar role as the noise sources above. There are factors that influence these variations for both color channels and may thus lead to correlated errors, such as variations in local chemical environment, illumination power, and missed localizations due to overlapping emissions in high density regions. In practice, the latter will usually have the largest impact, whereas the other factors are usually not very substantial. However, all these effects would only lead increase the apparent colocalization of both color channels, because they affect the density of localization in space. The orientation estimation would not be affected much, so no spurious co-orientation observations would be produced.

Stochasticity in labelling The process of fluorescently labelling a sample is another process that introduces stochasticity in the image formation. Firstly, there is stochasticity in the number of molecules that are labelled. For example, when fluorescent proteins are used then some of the proteins of interest will be of the wildtype variety without the fluorescent fusion protein. When antibody labelling is used, not all epitopes for the antibody will actually be bound by an antibody. Secondly, there is also stochasticity in the number of emitters associated with each of these markers such as antibodies: if secondary antibody labelling is used then the primary antibodies may have a varying number of secondary antibodies associated with them, and the number of emitters per secondary antibody may also vary.

If we make the reasonable assumption that these sources of stochasticity are uncorrelated between the molecules imaged in the different channels, then $c(\Delta\vec{x},\Delta\phi)$ remains unaffected: although the expected values of I_1 and I_2 are affected by these sources of stochasticity, they have a more or less equal effect on the numerator and denominator of $c(\Delta\vec{x},\Delta\phi)$ and therefore do not affect its expected value. The variance in the measurement of $c(\Delta\vec{x},\Delta\phi)$ may be substantially altered by these noise sources though.

References

References

1. Hell SW, Wichmann J. Breaking the diffraction limit resolution by stimulated emission: stimulated-emission-depletion microscopy. Opt Lett. 1994;19(11):780–783.

2. Klar TA, Jakobs S, Dyba S, Egner A, Hell SW. Fluorescence microscopy with diffraction resolution barrier broken by stimulated emission. Proc Natl Acad Sci U S A. 2000;97(15):8206–8210.

3. Heintzmann R, Cremer C. Laterally modulated excitation microscopy: improvement of resolution by using a diffraction grating. In: Bigio IJ, Schneckenburger H, Slavik J, Svanberg K, Viallet PM,

editors. Optical Biopsies and Microscopic Techniques III. SPIE Conference vol. 3568. Stockholm, Sweden; 1999. Doi:10.1117/12.336833.

4. Gustafsson MGL. Surpassing the lateral resolution limit by a factor of two using structured illumination microscopy. J Microsc. 2000;198(2):82–87.

5. Nieuwenhuizen RPJ, Lidke KA, Bates M, Leyton Puig D, Grünwald D, Stallinga S, et al. Measuring Image Resolution in Optical Nanoscopy. Nat Methods. 2013;10(6):557–562.
